# Harnessing Solute Carrier Transporters for Precision Oncology

**DOI:** 10.3390/molecules22040539

**Published:** 2017-03-28

**Authors:** Michael D. Nyquist, Bhagwat Prasad, Elahe A. Mostaghel

**Affiliations:** 1 Division of Human Biology, Fred Hutchinson Cancer Research Center, Seattle, WA 98109, USA; mnyquist@fredhutch.org; 2 Department of Pharmaceutics, University of Washington, Seattle, WA 98195, USA; bhagwat@uw.edu; 3 Division of Oncology, Department of Medicine, University of Washington, Seattle, WA 98195, USA; 4 Clinical Research Division, Fred Hutchinson Cancer Research Center, Seattle, WA 98109, USA

**Keywords:** solute carrier transporters, drug transport, chemotherapy, precision medicine, targeted therapy, SLC35F2, Survivin, YM155, precision oncology

## Abstract

Solute Carrier (SLC) transporters are a large superfamily of transmembrane carriers involved in the regulated transport of metabolites, nutrients, ions and drugs across cellular membranes. A subset of these solute carriers play a significant role in the cellular uptake of many cancer therapeutics, ranging from chemotherapeutics such as antimetabolites, topoisomerase inhibitors, platinum-based drugs and taxanes to targeted therapies such as tyrosine kinase inhibitors. SLC transporters are co-expressed in groups and patterns across normal tissues, suggesting they may comprise a coordinated regulatory circuit serving to mediate normal tissue functions. In cancer however, there are dramatic changes in expression patterns of SLC transporters. This frequently serves to feed the increased metabolic demands of the tumor cell for amino acids, nucleotides and other metabolites, but also presents a therapeutic opportunity, as increased transporter expression may serve to increase intracellular concentrations of substrate drugs. In this review, we examine the regulation of drug transporters in cancer and how this impacts therapy response, and discuss novel approaches to targeting therapies to specific cancers via tumor-specific aberrations in transporter expression. We propose that among the oncogenic changes in SLC transporter expression there exist emergent vulnerabilities that can be exploited therapeutically, extending the application of precision medicine from tumor-specific drug targets to tumor-specific determinants of drug uptake.

## 1. Introduction

The question put forward by this review is whether solute carrier (SLC) transporters can be used to target substrate drugs to cancers. While gains have been made in prolonging survival, cures for metastatic cancer largely do not exist (testis cancer being a notable exception). SLC transporters are a large superfamily of transmembrane carriers that move metabolites, ions, and drugs across cellular membranes. There are over 400 SLC transporters in 52 subfamilies grouped by sequence similarity [1]. For its size, the SLC transporter superfamily is one of the least studied groups of proteins [2]. To date, difficulties resolving crystal structures, toxicities associated with ectopic overexpression, overlapping substrate specificities, substrate-dependent inhibition and non-selective or non-available antibodies are frequent challenges encountered in the field. Notably, SLC transporters are co-expressed in groups and patterns across normal tissues, suggesting they may comprise a coordinated regulatory circuit serving to mediate normal tissue functions. In cancer, however, there are dramatic changes in expression patterns of SLC transporters, even more so than in protein kinase co-expression patterns [2]. We posit that among these oncogenic changes in SLC transporter expression there exist emergent vulnerabilities that can be exploited therapeutically, extending the application of precision medicine from tumor-specific drug targets to tumor-specific determinants of drug uptake.

Therapeutic targeting of drugs to tumors is highly appealing, both for promoting on-target specificity and sparing healthy tissues. To date, this has been approached by the use of antibody drug conjugates [3]. By contrast, exploiting a tumor’s intrinsic drug transport mechanisms to achieve targeted drug delivery is a novel and largely unexplored paradigm. This is partly due to the heterogeneity of SLC transporter expression in cancer, even within the same cancer type. However, a series of serendipitous discoveries demonstrating that androgen-regulated expression of *SLC35F2* is a key molecular determinant of response to the small molecule survivin inhibitor YM155 (Sepantronium Bromide) has shed light on new avenues of investigation [4,5].

We begin by briefly introducing the principle of carrier-mediated drug transport. We then review two important and clinically relevant classes of drugs, nucleoside analogs and tyrosine kinase inhibitors (TKIs), to illustrate how drug transporters are crucial determinants of therapy response, regardless of drug mechanism of action or target specificity. We then discuss potential strategies under development to “home” drugs to tumor cells by targeting aberrantly expressed or activated SLC transporters.

## 2. Carrier Mediated Drug Transport and Tumor Uptake: The Dominant Role of SLC22/SLCO Family Transporters

SLC22 and solute carrier organic anion (SLCO) family members are drug uptake carriers that play a significant role in nearly all pharmacological cancer treatments from antimetabolites and topoisomerase inhibitors to platinum-based drugs and taxanes [6,7,8]. These two SLC families are among the best described and understood due to their importance (along with the multidrug and toxin extrusion (MATE) and ATP-binding cassette (ABC) transporter families) in the pharmacokinetics of numerous drugs, metabolites, and nutrients [2]. In general, SLC22/SLCO solute carriers are highly expressed in tissues such as kidney, liver and intestine that are responsible for the absorption, metabolism and elimination of drugs and metabolites. They are also broadly expressed, at variable levels, in diverse organs and tissues throughout the body such as heart, brain, lung, placenta, salivary gland and testes [8,9]. Important examples include SLC22A1/OCT1, SLC22A2/OCT2, SLC22A4/OCTN1, SLCO1B1, SCLO2B1 and SLCO1B3 transporters, which have broad substrate specificity and mediate transport of numerous anti-cancer compounds such as irinotecan, paclitaxel, mitoxantrone, vincristine, methotrexate, 5-fluorouracil, platinum-based drugs, imantinib and doxorubicin, reviewed extensively elsewhere [1,2,10,11,12,13,14,15]. Variability in the expression or single nucleotide polymorphism (SNP) status of SLC22 and SLCO transporters by tumors can also be a significant determinant of drug sensitivity [10]. Here we briefly discuss the nucleoside family of SLC transporters to illustrate the importance of tumoral SLC transporter expression in drug response.

## 3. Nucleoside Transporters and Nucleoside Antimetabolites

Since the approval of mercaptopurine by the United States Food and Drug Administration (FDA) in 1953, nucleobase and nucleoside antimetabolites have been some of the most extensively studied families of anti-cancer drugs; a nucleotide consists of a nitrogenous base (the nucleobase), sugar and phosphate, while a nucleoside is only the nucleobase and sugar. The long history of research into the determinants of response and resistance to these drugs serves as a useful model for understanding the complexities of anti-cancer therapies [16]. While the specifics are not necessarily generalizable, determinants of response to nucleotide drugs illustrate key components of therapeutic efficacy: (i) drugs enter the tumor cells via specific SLC transporters; (ii) SLC transporter expression levels and function are major determinants of drug activity; and (iii) cancers may acquire resistance to drugs by reducing intratumoral drug concentrations via modulation of metabolic enzymes, downregulation of uptake transporters, or upregulation of ABC efflux transporters such as Multi-Drug Resistance Gene (MDR1/ABCB1).

Nucleoside family transporters mediate the uptake and exchange of nucleosides as well as nucleoside antimetabolite drugs (e.g., gemcitabine and cytarabine). Once inside cells, nucleosides and antimetabolite analogs are modified by a series of kinases and enzymes, such as deoxycytidine kinase (DCK), that are part of the nucleotide salvage pathways, resulting in the generation of tri-phosphate nucleotides. Processed nucleoside antimetabolites can disrupt enzymatic reactions such as nucleotide metabolism, polymerization, phosphorylation and methylation, as well as become incorporated into DNA causing DNA damage [17,18]. Nucleosides and their therapeutic analogs are transported into cells by two SLC protein families: SLC28 (SLC28A1, SLC28A2, and SLC28A3) and SLC29 (SLC29A1, SLC29A2, SLC29A3, and SLC29A4). The SLC29, or equilibrative nucleoside transporter (ENT), family members mediate the facilitated bidirectional exchange of nucleosides and their analogs. ENT1 and ENT2 can also transport nucleobases and therapeutic analogs such as 5-fluorouracil and 6-mercaptopurine [19]. The SLC28 or concentrative nucleoside transporter (CNT) family encodes cation-coupled nucleoside symporters. CNT1 and CNT2 are sodium ion-coupled symporters while CNT3 is coupled in a 2:1 cation-to-nucleoside ratio with sodium and proton ions, allowing nucleosides and nucleoside antimetabolites to be transported against their concentration gradient [19].

Although primarily concentrated in the polarized epithelia of the intestine, kidney, liver and brain [20], CNTs and ENTs are broadly expressed in tissues, and levels vary across and within cancers [19]. In clinical studies of gemcitabine in pancreatic and bladder cancers, tumor ENT1 expression correlates directly with overall survival and inversely with early relapse [6,16,20,21,22]. The ability of CNT3 to transport most nucleoside analogs using H^+^/Na^+^ ions symport marks it as another potential tumoral biomarker of sensitivity to nucleoside drugs [20,23,24]. Polymorphisms in nucleoside transporters that alter expression and/or transport efficiency also affect the tolerability and efficacy of nucleoside analogs [6,16,18,19,20,25].

Many of the main causes of therapy resistance to nucleoside drugs revolve around reducing the intracellular concentration of active drug by such mechanisms as downregulation of ENT1, mutation of drug-activating salvage enzymes like DCK, and increased expression of ABC family drug efflux transporters [17,26]. It is no surprise then that therapeutic drug monitoring (TDM) and dose adjustment to fit a therapeutic window is an effective approach to improving response and ameliorating systemic toxicities [27,28,29].

## 4. Membrane Transporters and Tyrosine Kinase Inhibitors

In contrast to nucleoside transporters, tyrosine kinase inhibitors (TKIs) are “targeted” to specific oncogenic signaling pathways that are critical for tumor growth and survival. Compared to chemotherapies, TKIs are anticipated to be better tolerated with reduced toxicities in normal tissues. In some cases, this appears to be true [30]. Unfortunately, in many cases, adverse reactions and complications frequently arise and can be just as severe as those of cytotoxic agents, causing some to look to the emerging role of drug transporters in TKI response for answers [31,32].

Beginning with the FDA approval of imatinib in 2001 there have been over two dozen TKI drugs approved for use against cancer. The ability of one TKI drug to inhibit multiple cellular kinases allows simultaneous targeting of redundant and cross-talking signaling pathways. However, despite the groundbreaking success of imatinib for treating chronic myeloid leukemia (CML), TKIs applied to many other cancers have had more moderate success [33]. Why many cancers respond poorly and/or exhibit de novo resistance to TKIs is still unexplained. While it is possible the optimal combination of kinase targets has yet to be identified, systemic and tumoral mechanisms of drug transport have been implicated as determinants of response (or lack thereof) to TKIs.

In vitro and in vivo preclinical models have identified some of the transporters that mediate the absorption, disposition and elimination of TKIs and their metabolized derivatives. While the exact role of specific transporters may be controversial due to methodological differences, in general, TKIs are effluxed by ABCB1 and/or ABCG2, and uptaken by SLCO and SLC22 transporters. An extensive review of TKIs and their transporters is covered by Neul et al. [33]. Mouse studies confirm the role of ABCB1 and ABCG2 in the efflux of TKIs [34]. Of note, the increase in intracellular accumulation of TKIs in SLC receptor overexpression models is usually less than 2-fold [33]. Numerous SNPs and halplotypes of transporter genes have been correlated with outcomes as well as adverse reactions to TKIs [32,33,35]. As examples, a high rate of loss of response to imatinib in CML was associated with the SLC22A1/OCT1 variant rs683369 (C490G) [36] and increased time to progression of gastrointestinal stromal tumors (GISTs) on imatinib was associated with the SLC22A4/OCTN1 variant rs1050152 (C1507T) [37]. Despite standardized dosing, absorption, distribution and metabolism of orally administered TKIs are affected by a host of environmental, systemic and genetic factors before the drug even reaches the tumor [6,33], such that intracellular and systemic TKI levels can vary widely, affecting both tumor response and the incidence of dose limiting toxicities [32,33,38,39,40,41]. Thus, despite the “targeted” nature of TKIs, many of the same considerations that determine the efficacy of “non-targeted” nucleoside analogs, with regards to systemic and tumoral exposure and resultant impacts on toxicity and efficacy, also apply to TKIs.

## 5. Rationale for Alternative Strategies

Cancer cells have numerous strategies for narrowing or closing the therapeutic window between antitumor efficacy and systemic toxicity, requiring novel strategies and therapeutic modalities that can address and overcome these resistance mechanisms. Lessons learned from nucleoside analogs illustrate the importance of intracellular drug accumulation and the various mechanisms by which cancer cells resist that accumulation. Even “targeted” therapies like TKIs rely on achieving threshold intratumoral levels as evidenced by pharmacokinetic and pharmacogenomics studies [27,41,42].

Given that small-molecule anti-cancer therapies require a threshold of intracellular accumulation to mediate cell death, an attractive strategy for treating cancer would be to somehow increase drug concentrations selectively in cancer cells. Unfortunately, the dominant transporters involved in drug uptake by cancer cells (e.g., SLC29s, SLC22s and SLCOs) have a limited ability to concentrate drugs intracellularly and are also highly expressed in the tissues responsible for the absorption, distribution, metabolism and elimination (ADME) of drugs. Similarly, inhibition of ABC family drug efflux transporters has also been challenging due to ubiquitous expression of these proteins and toxicities associated with perturbing ADME processes [43]. For these reasons, identifying tumor-selective transporters and developing drugs targeted to these mechanisms would be of considerable interest.

## 6. Exploiting Cation Coupled Drug Transport in the Tumor Microenvironment

Achieving high intracellular drug concentrations in a tumor-specific manner may be possible by hijacking the ability of SLC transporters to transport metabolites against their concentration gradient [44]. Proton gradient pumps are essential for maintaining the electrochemical potentials of organelles such as lysosomes, secretory vesicles and mitochondria [45]. SLC family members commonly use electrochemical gradients including proton- and sodium-coupled symport/antiport mechanisms to drive the uptake of numerous metabolites and xenobiotics against a concentration gradient including amino acids, peptides, vitamins, metals, salts, nucleic acids, drugs and environmental toxins [46].

Notably, the alterations in tumor metabolism, known as the Warburg effect, results in high rates of glycolysis and lactic acid secretion [47,48] such that the extracellular pH in the tumor microenvironment is often acidic compared to normal tissues [49]. Not only does this create the membrane potential to promote proton-coupled transport across the cancer cell membrane, but the acidic pH also broadens the substrate specificity of some proton-coupled transporters in the organic anion-transporting polypeptide (OATP/SLCO) family and proton-coupled folate transporter, PCFT (SLC46A1) [44,46].

In an intriguing example designed to exploit this phenomenon, investigators modified the structure of pemetrexed (PMX), a folate antimetabolite used in treatment of lung and bladder cancer, to favor uptake by the proton-coupled folate transporter (PCFT/ SLC46A1) [50]. Antifolates such as PMX and methotrexate are primarily transported by the reduced folate carrier (RFC/SLC19A1), which is widely expressed and is the preferred transport mechanism at physiological pH. However, folates are also transported by PCFT, which is preferentially active at acidic pH. Thus, in the acidic microenvironment of the tumor, PMX derivatives that are selectively transported by PCFT may result in tumor-specific uptake.

While promising, compounds with pH-regulated tumoral uptake have yet to be proven in a clinical setting, and it is unlikely that disseminated tumor cells are able to generate a similarly acidic microenvironment. Thus, an attractive alternative would be drugs that can target a concentrative receptor that is already aberrantly expressed by cancer versus normal tissues.

## 7. Exploiting Tumor Dependency on Amino Acid and Peptide Transport

Tumor cells are particularly dependent on amino acid transport to support their increased energetic and metabolic needs, providing an attractive target of opportunity for drug delivery [48,51,52,53]. Membrane- and organelle-based transporters are also critical for the sensing the metabolic environment of the cell and regulating critical oncogenic pathways such as mammalian target of rapamycin (mTOR) signaling [54,55]. For example, SLC1A5 takes up l-glutamine which is then exchanged for mTOR-activating l-leucine and other essential amino acids via the bidirectional antiporter SLC7A5, and both SLC1A5 and SLC7A5 are overexpressed in cancers along with glutamine transporter, SLC6A14 [52].

There are a multitude of amino acid transporters spanning 11 SLC gene families that traffic amino acids from the extracellular environment into the cytoplasm or from the cytoplasm into the lumens of organelles. These transporters recognize diverse amino acid substrates and usually either exchange one amino acid for another or utilize Na^+^ gradients to uptake amino acids. The latter mechanism, of which the sodium/chloride-coupled transport of SLC6A14 is an example, imparts a high capacity to transport substrates against their concentration gradient and allow accumulation in the cell [52,56]. Many cancers, particularly those of epithelial origin such as cervical [57], pancreatic ductal adenocarcinoma [58] and breast cancer [59] upregulate SLC6A14 to take advantage of its high capacity for glutamine uptake [52]. Development of amino acid-based prodrugs that are recognized as substrates by SLC6A14 is an emerging strategy designed to exploit this vulnerability and target drugs to cancer cells with upregulated SLC6A14 [56]. Once transported inside the cell, these drugs are metabolized by endogenous esterases to the active form [56,60]. Similar peptide conjugated prodrug strategies are being pursued to target cancer compounds to proton-coupled peptide transporter PEPT1/SLC15A1, which is overexpressed by some prostate cancers [61,62,63].

## 8. Targeting Drugs to Cancers: YM155 and the Nucleotide Transporter SLC35F2

In this section, we discuss an evolving story surrounding the survivin inhibitor YM155 and recent studies demonstrating that regulation of its transporter, the nucleotide transporter SLC35F2 is, at least in part, regulated by androgen receptor signaling. YM155 exemplifies several aspects of a drug that is likely to have a clinically relevant transport-dependence: (1) it targets core aspects of tumor cell growth and survival; (2) it is a direct target/substrate of an aberrantly expressed SLC transporter; and (3) it has potential for clinical development as a biomarker-reliant drug.

### 8.1. The Preclinical Potential of YM155

Survivin (BIRC5) is an oncogene that imparts increased growth and resistance to apoptosis and is expressed selectively in cancers and undifferentiated cells but not normal tissues [64]. YM155 was identified from drug screens designed to find small molecules that can suppress the transcription of survivin, as survivin itself is not highly druggable [65,66,67]. Subsequent preclinical evaluation showed YM155 suppressed the viability of over one hundred cell lines, regardless of p53 status, and was associated with high efficacy and low systemic toxicity in xenografts of multiple tumor types [67,68,69,70].

While controversial, mechanistic studies establish that the cellular effects of YM155 reach beyond survivin suppression alone [71]. YM155 can disrupt the DNA-binding of transcription factors SP1 [72], ILF3 [73], p50 [74] and NONO [75], and suppress the expression of *XIAP*, *Mcl1*, *Sox2*, *Bcl-2* and *Bcl-xl* [74,76], mediating autophagy-dependent apoptosis [77,78] and DNA damage [5,78,79]. Given their importance regulating survival and stem cell signaling, targeting of these factors may explain how YM155 can selectively kill cancer and teratoma-forming cells while sparing differentiated tissues [71,80].

### 8.2. Mixed Clinical Results: Disappointment and Opportunity

While preclinical studies of YM155 were very encouraging, YM155 has been only modestly effective in phase I and II clinical trials. While YM155 was well tolerated, responses in a phase I pharmacokinetic trial of YM155 in 41 patients with advanced cancers were limited, including prostate specific antigen (PSA) responses in two prostate cancer patients and one complete and two partial responses in three patients with non-Hodgkin’s lymphoma [81]. In a phase II study of YM155 in 35 docetaxel-refractory castration resistant prostate cancer patients, single-agent therapy with YM155 achieved prolonged stable disease of ≥18 weeks in 25% of patients [82]. Other phase II studies of YM155 monotherapy in treatment refractory diffuse large B-cell lymphoma [83] and non-small cell lung cancer [84] showed 7.3% and 5.4% of patients achieved partial or complete responses, respectively. Studies paring paclitaxel or docetaxel with YM155 did not show added benefit for YM155 as a co-therapy in melanoma [85], human epidermal growth factor receptor 2 (HER2)-negative metastatic breast cancer [86] and non-small-cell lung cancer [87].

### 8.3. SLC and ABC Transporter Expression Predicts Response to YM155

Importantly, in light of the observation that YM155 was only effective in a subset of patient, preclinical data have shown that expression of the orphan nucleotide transporter SLC35F2 and the multidrug resistance transporter ABCB1 are major determinants of response to YM155. SLC35F2 was discovered to transport YM155 using a retrovirus gene trap screen on the near haploid cell line KBM7 [5]. Sensitivity to YM155 was correlated with SLC35F2 expression across a wide range of cell lines from various cancer types [5,88]. YM155 is also a substrate of SLC22A1 and SLC22A2 [89]. By contrast, resistance to YM155 in neuroblastoma was shown to be mediated by high expression of the MDR1/ABCB1 exporter and thus ABCB1 is also major determinant of YM155 sensitivity [90,91,92].

### 8.4. Nucleotide Transporter SLC35F2—Mediator of Intracellular YM155 Accumulation

Post-translational modification of protein, lipids and proteoglycans by glycosylation is a critical cellular process that becomes dysregulated in cancer [93,94]. Glycosylation regulates the trafficking, solubility, stability and extracellular interactions between membrane and secreted proteins as well as nuclear and cytoplasmic proteins. While complex glycosylations of proteins occur in the endoplasmic reticulum and Golgi, nucleotide sugars are synthesized in the cytosol and must be transported into these organelles by the SLC35 family of nucleotide transporters. Mutations and deficiencies of nucleotide transporters are causal in numerous developmental diseases related to defects in protein trafficking and regulation as well as endoplasmic reticulum (ER) stress [95,96,97,98].

Seven subfamilies of the nucleotide sugar transporters have been identified including SLC35A–G, with E–G being “orphan” transporters without known physiological roles and substrates. Known SLC35 family members use an antiport mechanisms to transport a nucleotide diphosphate-linked sugar from the cytosol into the lumen of the organelle in exchange for a nucleotide monophosphate [96]. Transport is a time-dependent and saturable process that is able to concentrate nucleotide sugars within the lumen of the ER or Golgi. Transport can be inhibited by the presence of mono- and diphosphorylated nucleosides, which seem to be the determining element for SLC substrate specificity [96,97,98].

Consistent with the concentrative, antiport mechanism of SLC35-nucleotide family transporters [96], tumor models with high levels of SLC35F2, such as the PC3 prostate cancer cell line [5], are able to accumulate high intratumoral levels of YM155. Accordingly, YM155 levels in PC3 xenografts were 20-fold higher than YM155 levels in serum [67]. A subsequent study using radioactively labeled YM155 found even higher tumor YM155 levels, with a tumor to serum uptake ratio of 26.5 (±2.9) and tumor-to-muscle ratio of 25.6 (±3.6) [99]. These studies suggest that the efficacy of YM155 is likely critically dependent on intracellular concentrations, as seen with the nucleoside analogs, yet is cancer-selective in mediating cell death, as seen with TKIs.

### 8.5. Pharmacologic Induction of SLC35F2 and Sensitivity to YM155

A recently published study by our group found that SLC35F2 is regulated by the androgen receptor (AR). The emerging role of high-testosterone (T) therapy in prostate cancer makes AR-induced sensitivity to SLC35F2 transported therapeutics clinically relevant [100,101]. AR activity can be dichotomous in action by promoting prostate cancer growth under normal circumstances and retarding its growth when overstimulated with excessive androgens [102]. Acutely heightened AR signaling induces cell stress and cell cycle arrest by upregulating negative regulators of the cell cycle [103,104] and inducing DNA topoisomerase 2-beta (TOP2B)-mediated double-strand breaks in DNA [105].

Using high-throughput drug screening, we found that YM155 synergized with high-dose androgen therapy in prostate cancer cells, and that this interaction was mediated by direct transcriptional upregulation of SLC35F2 by AR [4]. Ectopic overexpression of SLC35F2 limited the ability of androgens to sensitize cells to YM155, suggesting the effect is saturable, while overexpression of ABCB1 completely blocked androgen-mediated YM155-induced cell death. In patient-derived xenografts (PDX) models of advanced prostate tumors, SLC35F2 expression was correlated with intratumoral androgen levels in three models and with expression of constitutively active AR splice variants in a fourth, while androgen-withdrawal via castration of tumor-bearing mice reduced SLC35F2 expression, all consistent with regulation of SLC35F2 by AR axis signaling. Analysis of ~150 castration-resistant prostate cancer metastases revealed that SLC35F2 expression correlated with AR activity score, but that a subset of patient tumors had high expression of the transporter regardless of AR status [4].

### 8.6. Re-Examining Clinical Trials: SLC35F2 and ABCB1 as Biomarkers of Response to YM155

These emerging data on the molecular determinants of YM155 sensitivity suggest that the subset of patients that respond to YM155 in clinical trials are likely those with high SLC35F2 expression and low ABCB1 expression. In particular, eligibility requirements for the prostate cancer trial [82] required castrate levels of serum testosterone (≤50 ng/mL), and decreased AR-activity may have caused a downregulation of SLC35F2 that prevented effective tumor uptake of YM155. Thus, high-dose testosterone therapy may enhance sensitivity to YM155 in castrate prostate cancer patients with low tumoral SLC35F2 expression by inducing SLC35F2, while high SLC35F2 expression at baseline serving as biomarker to select patients who may be immediate candidates for YM155 treatment.

## 9. Oncogenic and Pharmacologic Regulation of SLC Transport Proteins

Tissue-selective SLC profiles reflect the specific metabolic needs and environmental cues that mediate normal cell growth and homeostasis [98,106]. Oncogenic transformation and accompanying metabolic alterations commonly result in aberrant expression of SLC transporters, including expression of drug transporters, which may influence therapeutic response. Interestingly, the SLC transporter expression profiles of cancer cell lines does not necessarily correspond to their tissue of origin [107], differing somewhat from profiling of ABC transporters in cancer [108]. Peptide transporters normally expressed in intestinal and renal epithelia are overexpressed in prostate cancer [62]. Prostate cancers also upregulate SLCO2B1 and SLCO1B3 [109,110,111], possibly to facilitate uptake of androgens from the serum. Folate and peptide transporters PCFT and PEPT1 are aberrantly expressed in many other tumors and cancer cell lines [44], as are the nucleotide transporters ENT1 and CNT3 [19].

The causal mechanisms of upregulation, and reasons for variability are not well understood. However, several links have been established between oncogenic signaling pathways and changes in transporter expression. Both oncogene overexpression and tumor suppressor loss have been implicated in altering SLC transporter expression as part of a coordinated growth program, particularly in the highly important metabolic processes of glutamine and amino acid metabolism. In this regard, amino acid transporters SLC1A5 and SLC38A5 are c-Myc targets [112,113,114]. SLC1A5 is also upregulated by Retinoblastoma protein (RB1) loss and subsequent transcription factor E2F1 signaling [115]. The peptide transporters PEPT1 and PEPT2 are upregulated by Janus Kinase JAK2 [116]. Finally, ENT1 was found to be upregulated during cell cycle [19]. 

A potentially important ramification of oncogenic regulation of SLC transporters is that anti-cancer therapies may reverse this upregulation, causing a therapeutic desensitization of the tissues. For example, TKIs can inhibit expression of OATP1B1 and OATP1B3, which are important uptake mechanisms [6,117], as well as inhibiting expression of nucleotide transporters, potentially explaining why combination therapies using gemcitabine and TKI have not succeeded [118].

Hormones can affect SLC transporter expression [119] and may provide an avenue for pharmacologic induction of tumoral SLC expression. As discussed earlier, the amino acid transporter SLC6A14 is directly upregulated by estrogen/ER activity in ER^+^ breast cancers [60]. Potentially, a subset of cancers with ER mutations leading to persistent ER activity driving SLC6A14 expression might be targeted or identified using this transporter [120]. Similarly, we have recently shown that SLC35F2 is a direct transcriptional target of AR, and that its expression in human prostate tumors is correlated with AR activity and tumor androgen levels.

The extent to which other members of the SLC35 nucleotide transporter family mediate uptake of other oncology compounds, are subject to hormonal regulation, or change with oncogenic transformation (potentially in conjunction with cancer-related changes in glycosylation) is unknown. Many SLC35 family members exhibit tissue specific in expression [106]. SLC35F2 appears to be preferentially expressed in the salivary gland, with lower, but as we have shown, AR regulated-expression in prostate [106]. Interestingly, SLC35A1, which transports cytidine monophosphate (CMP)-sialic acid, is most highly expressed in normal prostate [106], and the glycosylation of PSA, which is modified by sialic acid sugars, changes in prostate cancer [121]. SLC35F2, also known as lung squamous cell cancer related protein (LSCC-3), is also upregulated in non-small cell lung cancer [122] with knockdown of SLC35F2 in lung cancer cell lines reducing proliferation and invasion, although whether this relates to nucleotide transport is unknown [123]. While no other SLC35 transporters are known to transport oncology compounds, one study correlated SLC35A5 expression to paclitaxel sensitivity [124]. In order to unlock the potential of SLC35 transporters as drug transporters, further research is needed to identify endogenous and synthetic substrates as well as to delineate the structural basis of substrate recognition.

## 10. Future Perspectives: a Potential Precision Oncology Approach to Exploiting Changes in SLC Expression Profiles

Cytotoxic chemotherapy is characterized by non-specific targeting of rapidly dividing cells and often has a narrow therapeutic index [29], while precision oncology refers to drugs that target cancer-specific signaling pathways, holding the promise of enhanced efficacy and decreased toxicity. However, both chemotherapeutics and molecularly targeted drugs can be markedly influenced by drug uptake and export mechanisms that modulate tumor-level drug exposure. Tumoral expression of the ABC/MDR family of drug exporters is a well-recognized mechanism of resistance to cancer therapeutics. Similarly, renal and hepatic expression of SLC transporters is well recognized as an important determinant of systemic drug exposure and metabolism. However, tumor-specific expression of concentrative SLC transporters is likely to be an equally important mediator of drug sensitivity, as well as representing an under-explored therapeutic opportunity for targeting drugs to tumor tissues. Taxane transport by SLCO/SLC22 transporters is an important example of the former phenomenon. Increased hepatic expression of OAT2 (SLC22A7) in the castrate setting has been linked to increased drug clearance and decreased toxicity in castrate men with prostate cancer receiving docetaxel compared to non-castrated patients or patients with other solid tumors (reviewed by Sprowl et al. [125]), while loss of tumoral OATPB3 (SLCO1B3) expression was linked with resistance to therapy and decreased intracellular concentrations of docetaxel in taxane-resistant models of prostate cancer in vivo [126]. A clinically relevant example of manipulating transporter expression for therapeutic drug targeting that has been successfully taken into phase I studies is the use of histone deacetylase inhibitor voronistat to enhance expression of the norepinephrine transporter (NET/SLC6A2). NET is expressed on neuroblastoma cells and is the uptake transporter for the norepinephrine analog meta-iodobenzylguanidine (MIBG), a key therapeutic in the treatment of neuroblastoma [127,128].

Further research to identify endogenous and synthetic substrates of under-characterized SLC family members, delineate the structural basis of substrate recognition, and determine factors regulating transporter expression will be critical to identify specific transporters that may serve as biomarkers of response and resistance, as well as to reveal novel opportunities for pharmacologic regulation of tumor drug targeting. Due to the variability of SLC expression in cancer, use of “transportome” profiling to predict drug response requires direct measurements of tumor transporter expression and genetic characteristics. Transporter proteomics is emerging as a vital technique to quantitatively profile levels of these proteins in cells and tissues. Using proteomic approaches, not only can transporters be quantified at absolute levels but determinations of their subcellular localization and regulation, including posttranslational modification can also be achieved [129,130]. Establishing a therapeutic biomarker based on transporter expression, protein level, or SNP status for drug sensitivity can be folded into the developing field of precision oncology but requires several conditions to be met; (1) Robust assays for characterizing RNA, protein [131] or DNA [132] in tissues; (2) a broad clinical sample set to make determinations about relative expression of a particular biomarker so that distinct patient cohorts can be identified; and (3) a complex set of preclinical models where relationships between drug response and biomarker characteristics can be investigated under a variety of circumstances. While difficult, developing such biomarker-driven clinical approaches for drug transporters as well as drug targets is critical to the success of precision medicine.
